# Vaccines for the 21st century

**DOI:** 10.1002/emmm.201403876

**Published:** 2014-05-06

**Authors:** Isabel Delany, Rino Rappuoli, Ennio De Gregorio

**Affiliations:** Novartis VaccinesSiena, Italy

**Keywords:** adjuvants, clinical trials, infectious diseases, structural vaccinology, vectors

## Abstract

In the last century, vaccination has been the most effective medical intervention to reduce death and morbidity caused by infectious diseases. It is believed that vaccines save at least 2–3 million lives per year worldwide. Smallpox has been eradicated and polio has almost disappeared worldwide through global vaccine campaigns. Most of the viral and bacterial infections that traditionally affected children have been drastically reduced thanks to national immunization programs in developed countries. However, many diseases are not yet preventable by vaccination, and vaccines have not been fully exploited for target populations such as elderly and pregnant women. This review focuses on the state of the art of recent clinical trials of vaccines for major unmet medical needs such as HIV, malaria, TB, and cancer. In addition, we describe the innovative technologies currently used in vaccine research and development including adjuvants, vectors, nucleic acid vaccines, and structure-based antigen design. The hope is that thanks to these technologies, more diseases will be addressed in the 21st century by novel preventative and therapeutic vaccines.

## Introduction

**Figure 1:**
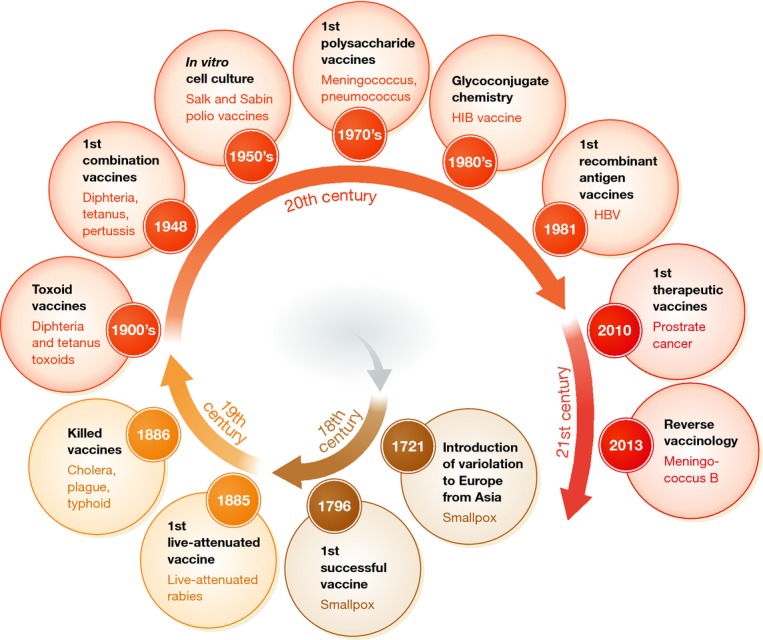
Major milestones in the historical path of the development of vaccinology and vaccine design A method for preventing naturally acquired smallpox called ‘variolation’ was discovered in India before 1,000 A.D. and was in use also in China and Western Asia. This method, which consisted of the inoculation of pustule material from smallpox-infected patients to healthy individuals, was introduced in Europe in 1,721 by Lady Mary Wortley Montagu. The first real vaccination practice was introduced when Edward Jenner used pustule material from humans infected by cowpox to protect against smallpox.

Progress in science has always been the major driving force for development of effective vaccines (Fig 1). Table 1 provides a list of all licensed human vaccines, grouped in different classes based on the method of production (reviewed in Plotkin *et al*, 2008; Levine *et al*, 2012; De Gregorio *et al*, 2013). The first golden age of vaccines started when Pasteur, Koch, Ramon, and Mérieux established the germ theory and developed vaccines based on live-attenuated or inactivated (killed) pathogens and on inactivated toxins (toxoids). These vaccines protected against rabies, diphtheria, tetanus, pertussis, and tuberculosis in infants. The second golden age of vaccines was a consequence of innovation in cell culture technologies in the second half of the 20th century. The ‘cell culture revolution’ allowed for effective inactivated vaccines to prevent polio (IPV) and hepatitis A, and live-attenuated vaccines against polio (OPV), mumps, rubella, measles (MMR), rotavirus, and varicella. Progress in microbiology led to the development of polysaccharide vaccines against some strains of pneumococcus and meningococcus. However, these vaccines were not effective in children. To improve immunogenicity, the antigenic polysaccharides, which primarily induce a B-cell-dependent immune response, were covalently linked to carrier proteins, thereby providing helper T-cell activation. The resulting glycoconjugate vaccines induced a better antibody response and were effective in all age groups. Today, very effective glycoconjugate vaccines are available for *Haemophilus influenzae*, pneumococcus, and the meningococcus types A, C, W, and Y. Hepatitis B virus (HBV) and human papillomavirus (HPV) cannot be easily cultured *in vitro* for vaccine production, and the first-generation HBV vaccine consisted of purified HBV surface antigen from the blood of infected donors. Progress in molecular biology allowed the improvement of the vaccine against HBV and, more recently, the development of a new vaccine preventing HPV. Both vaccines are made of purified recombinant protein antigens that form a non-infectious viral-like particle (VLP). In the last decade, progress in genomics has also contributed to vaccine development. Unlike the other meningococci, *Neisseria meningitidis* type B (MenB) is covered by a capsular polysaccharide that is similar to polysaccharide present in human tissues and therefore poorly immunogenic. As such, the MenB capsular polysaccharide cannot be used in a glycoconjugate vaccine, unlike what was efficiently done for types A, C, W, and Y (Pace, 2009). Making a vaccine based on recombinant proteins was also challenging because of the extreme antigenic variation seen in circulating MenB strains. The problem was solved through a rational selection of candidate antigens based on genomic information, called ‘reverse vaccinology’ (Pizza *et al*, 2000; Rappuoli, 2000). Through this process, three protective antigens that are common to multiple MenB strains were expressed as recombinant proteins and combined with a MenB outer membrane vesicle (OMV), resulting in the first universal vaccine against type B meningococcus (Giuliani *et al*, 2006). All the vaccines described above are given to healthy subjects to prevent infections. In addition, some prevent cancer associated with chronic infection, HPV, and HBV (Pineau & Tiollais, 2010; Romanowski, 2011). The therapeutic use of vaccination based on specific antigens associated with the disease has not had equal success despite many attempts to cure chronic infections and cancer. However, in 2010, the FDA approved Sipuleucel-T, the first therapeutic vaccine for prostate cancer. The administration of Sipuleucel-T is very different from all licensed preventive vaccines. Blood cells from each individual prostate cancer patient are exposed to a prostate antigen (prostatic acid phosphatase) fused to the cytokine GM-CSF and then re-infused to the same patient (Plosker, 2011). Although the immunization process is very complex and expensive, Sipuleucel-T represents a milestone and may pave the way for a wider use of cancer vaccine immunotherapy based on innovative technologies that allow for simpler immunization methods. Several cancer vaccine candidates, based on recombinant antigens or viral vectors, are in advanced development with promising phase II results (Kruit *et al*, 2013). If they confirm their partial efficacy in larger phase III trials, the next step will be to combine cancer vaccines with additional immunotherapies such as monoclonal antibodies acting on negative regulators of the immune response (e.g., CTLA-4 and PD-1 Hodi *et al*, 2010; Hamid *et al*, 2013) recently described by *Science* as the ‘breakthrough of the year’ for 2013 (Couzin-Frankel, 2013).

**Glossary**

**Vaccine**

all biological preparations that enhance immunity against disease and either prevent (prophylactic vaccines) or treat disease (therapeutic vaccines): The word ‘vaccine’ originates from the Latin Variolae vaccinae (cowpox), which Edward Jenner demonstrated in 1798 could prevent smallpox in humans

**Immunotherapy**

treatment of disease by inducing, enhancing, or suppressing an immune response

**Live attenuated**

a viable infectious organism with reduced virulence or ability to cause disease: infectious agents may be attenuated by *in vitro* passage, chemically, genetically, or other means

**Inactivated**

a killed infectious organism: whole organisms may be inactivated by chemical, thermal, or other means

**Subunit vaccines**

vaccines derived from components of the disease-causing organism, such as specific proteins and polysaccharides

**Polysaccharide vaccines**

vaccine derived from the complex sugar capsular polysaccharide that covers the surface of encapsulated bacteria

**Conjugate vaccine**

vaccines derived from the covalent linkage (conjugation) of polysaccharides to a carrier protein for enhanced immunogenicity

**Combination vaccines**

vaccines against different disease-causing organisms combined into one formulation for a unique immunization

**Synthetic vaccines**

vaccines based on synthetic components such as nucleic acids or synthetic peptides, polysaccharides, or antigens

**Recombinant**

derived from the use of recombinant DNA technology

**Reverse vaccinology**

a method of producing a vaccine by first studying the genomic information of the organism (*in silico*) to determine which genes code for candidate antigenic proteins, followed by *in vitro* and *in vivo* testing of those candidates and selection for vaccine development

**Serotype**

the type of a microorganism determined by its constituent antigens

**Serogroups**

a group of serotypes having one or more antigens in common

**Neutralizing antibodies**

An antibody that reduces or abolishes some biological activity of a soluble antigen or of a living microorganism

**Opsonizing antibodies**

An antibody that causes bacteria or other foreign cells to become more susceptible to the action of phagocytes

**Nosocomially acquired antibiotic-resistant bacteria**

hospitally acquired bacteria that are no longer susceptible to treatment with antibiotics

**Table 1:** Licensed vaccines are grouped into seven classes based on the method of production: live attenuated, killed whole organisms, toxoids/proteins, polysaccharides, glycoconjugates, recombinant, and personalized blood cell re-infusion

Method of production	Licensed vaccines
Live attenuated	Smallpox, rabies, tuberculosis (BCG), yellow fever, polio (OPV), measles, mumps, rubella, typhoid, varicella, rotavirus, influenza (cold adapted), zoster

Killed whole organism	Typhoid, cholera, plague, pertussis, influenza, typhus, polio (IPV), rabies, Japanese encephalitis, tick-born encephalitis, hepatitis A

Toxoid/protein	Diphtheria, tetanus, acellular pertussis, anthrax, influenza subunit

Polysaccharide	Pneumococcus, meningococcus, Haemophilus influenzae B, typhoid (Vi)

Glycoconjugate	*Haemophilus influenzae* B; pneumococcus (7, 10, and 13 valent), meningococcus C, meningococcus ACWY

Recombinant	Hepatitis B, cholera toxin B, human papillomavirus; meningococcus B; hepatitis E

Blood cell infusion	Prostate cancer

In summary, the application of innovative technologies in the last century has allowed for the development of novel vaccines targeting new diseases or new target populations. In the next paragraphs of this review, we will focus on vaccines for the prevention of infectious diseases, give an overview of recent clinical trials of some important vaccine candidates in development, and discuss which target populations are not adequately protected by vaccines. Finally, we will assess the novel immunization technologies that can be developed today to address the medical needs of the 21st century (GIVS 2006–2015 at http://www.who.int/immunization/givs/en/).

## Medical needs and challenges

**Figure 2:**
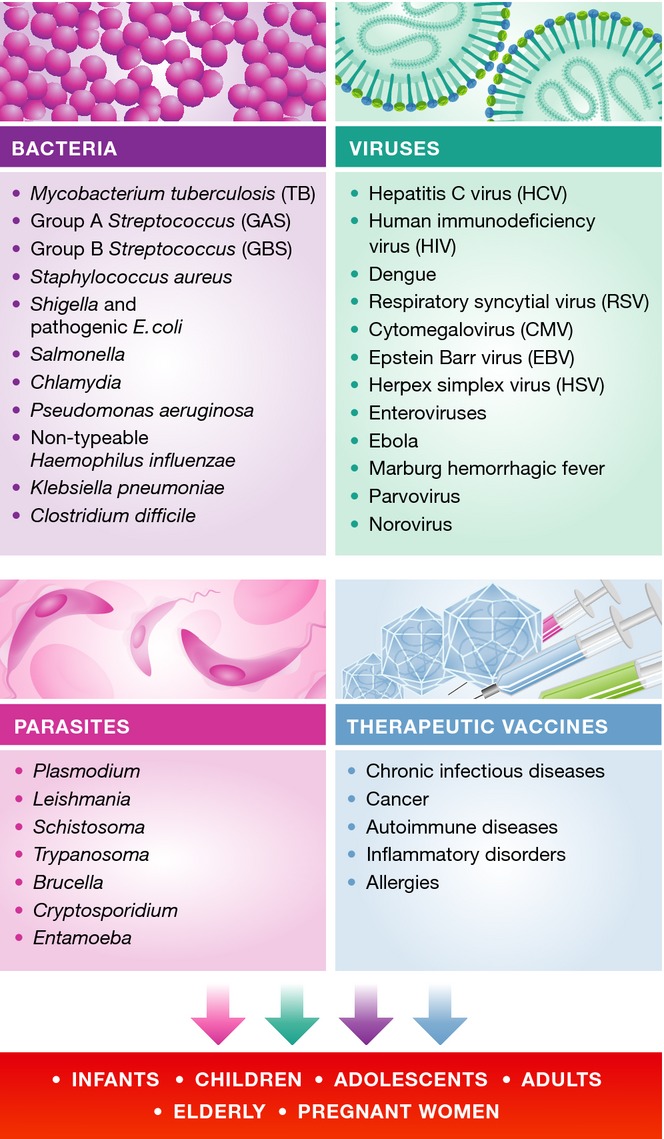
Target disease and target populations for 21st century vaccine development Included in the list are the agents of infectious diseases for which vaccines are not yet available or for which more effective vaccines would be beneficial. Also included are therapeutic vaccines for chronic infectious diseases, as well as non-communicable pathologies such as autoimmune diseases, cancer, and allergy, some of which are in advanced clinical trials.

Routine immunization programs protect most of the world's children from a number of infectious diseases that previously claimed millions of lives each year. For travelers, vaccination offers the possibility of avoiding a number of infectious diseases that may be encountered abroad. However, satisfactory vaccines have not yet been developed against several widespread and life-threatening infections. Human immunodeficiency virus (HIV) affects more than 30 million people worldwide (UNIAIDS Global Report at http://www.unaids.org/en), while malaria and tuberculosis kill almost 3 million people every year (WHO report 2010: http://www.who.int). Other examples of pathogens that may be prevented by vaccination and for which vaccines are not yet available are hepatitis C virus (HCV), dengue, respiratory syncytial virus (RSV), cytomegalovirus (CMV), group B *Streptococcus* (GBS), *Staphylococcus aureus,* and *Pseudomonas aeruginosa* (Fig 2).

Vaccines developed in the twentieth century have been effective in protecting against pathogens with a low degree of antigen variability. Pathogens that exist in multiple strains exhibiting a moderate degree of antigen variability require multivalent vaccines. The most successful example of this is possibly pneumococcus, for which a 7-valent and a 13-valent conjugate and a 23-valent polysaccharide vaccine have been developed covering a progressively broader number of serogroups (Prymula & Schuerman, 2009; Duggan, 2010). A more extreme situation exists for seasonal influenza vaccines (an organism which rapidly alters), which, while multivalent, must be redeveloped every year incorporating the influenza surface antigens of predicted circulating disease variants. However, until now, vaccines have not been successful in protecting against pathogens characterized by a high mutation rate, such as HIV and HCV that are able to evade the antibody response by modifying their target antigens during the course of infection. In addition, most licensed vaccines are believed to prevent infections by generating neutralizing or opsonizing antibodies. There is, nonetheless, a crucial contribution of T cells. For instance, T helper cells contribute to efficient B-cell activation, influence antibody isotype switching and activation of target cells (e.g., macrophages, neutrophils, and eosinophils). For example, differential induction of Th1/Th17 versus Th2 cells leads to improved protection in whole cell bacterial vaccines like pertussis (Ross *et al*, 2013). Also, a direct contribution of cellular immunity, in the form of cytotoxic CD8 and CD4 T cells, has been shown to play a role for live-attenuated vaccines. Conventional technologies have had only limited success in preventing infections that are controlled predominantly by T cells, such as tuberculosis. The challenge for the future is even greater, as some infections that are caused by highly variable pathogens may not be preventable by antibodies alone and will require the correct combination and quality of humoral and cellular immune responses.

For many pathogens, natural infection leads to immunity of the host against re-infection. Many highly successful vaccines, such as live-attenuated or inactivated vaccines, may rely on direct mimicry of the natural immunity induced by the pathogen. However, satisfactory vaccines have not yet been developed against infections that fail to elicit a protective immune response against the causative organism. For instance, for those diseases that do not induce sterilizing immunity after natural infection (e.g., malaria, RSV, or *P. aeruginosa*) or those that cause persistent or latent infection (e.g., HIV and HCV and *S. aureus*), a vaccine-induced protective immune response must go beyond the mechanisms that nature has evolved. Furthermore, the immune response against the determinants of certain viral agents, such as RSV or dengue virus, can actually exacerbate disease with low levels of antibody giving rise to enhancement of infection (Kim *et al*, 1969; Halstead, 1988).

Depending on the type of infection to be prevented, an effective vaccine may require the induction of different humoral and cellular immune effector mechanisms. A lack of understanding in the pathogenesis of the infecting organism, the absence of good animal models, and also the lack of correlates of protection are all factors that have contributed to the difficulties in developing some of the more challenging vaccines. Among them, and despite decades of concerted efforts in vaccine research, HIV, malaria, and tuberculosis represent diseases for which there are currently no highly effective candidate vaccines close to licensure. Recent failures in late-phase clinical trials highlight the difficulties that have been encountered.

## Selected clinical trials on vaccines for the prevention of infectious diseases

### HIV

HIV is the fourth largest killer in the world today with an annual death toll of approximately 2 million and over 7,000 new infections daily (Koff *et al*, 2013). While nearly three decades have passed since the identification of HIV as the causative organism of AIDS, attempts to develop effective vaccines against the highly variable retrovirus have been repeatedly stymied. The challenges of developing an HIV vaccine are multifold and include the global variability of HIV; the lack of a validated animal model, correlates of protective immunity, and of natural protective immune responses against HIV; the reservoir of infected cells conferred by integration of HIV's genome into the host; and the destruction of the immune cells by HIV infection. The driving forces in HIV vaccine design have moved from either targeting antibody responses with protein antigen vaccines or cell-mediated responses with viral vectors and gene-based vaccines, respectively, to vaccines which attempt to elicit both cellular and humoral immune responses with heterologous prime-boost regimens.

Initial HIV vaccine trials attempted to elicit protective antibody responses to soluble HIV-1 envelope protein (gp120), but failed to show any efficacy (Flynn *et al*, 2005; Pitisuttithum *et al*, 2006). Two clinical trials (STEP and Phambili) were conducted with the same candidate MRKAd5, a multivalent recombinant adenovirus vectors (rAd5) expressing multiple antigens (including clade B Gag, Pol, and Nef and lacking Env) intended to induce cellular responses. Despite the induction of HIV-1 Gag- and Pol-specific CD8^+^ T-cell responses in a majority of subjects, early viral loads were not decreased (Buchbinder *et al*, 2008; Gray *et al*, 2010, 2011). In addition, an increased risk of acquisition was observed in a subset of vaccinees with pre-existing Ad5 antibodies in the STEP trial (Buchbinder *et al*, 2008; McElrath *et al*, 2008). The recent failure and discontinuation of the HVTN505 efficacy trial represents another hard blow to HIV vaccine advancement. The trial used DNA prime and rAd5 vector boosts with multiple antigens (HIV-1 modified *env* genes from clades A, B, and C, and *gag* and *pol* genes from clade B) for elicitation of both antibody and T-cell responses and was performed on subjects without pre-existing antibodies against rAd5. This vaccine failed to show protection, and despite the preselection of rAd5 seronegative subjects, a trend toward more infections among the vaccinees was observed although not statistically significant (http://www.hvtn.org/505-announcement-25April2013.html). The lack of efficacy in this trial suggests that future HIV vaccine strategies should avoid human adenovirus-based vector approaches.

A more successful approach was based on the combination of two vaccines: a recombinant canarypox vector and an envelope (gp120) subunit in a prime-boost strategy. This vaccine was administered in Thailand in the so-called RV144 trial and protected 31.2% of the subjects from HIV acquisition (Rerks-Ngarm *et al*, 2009; Haynes *et al*, 2012). This study showed for the first time that prevention of HIV infection can be achieved through vaccination. Follow-up studies showed that antibodies directed against the V1–V2 variable regions of envelope gp120 correlated inversely with infection risk (Haynes *et al*, 2012).

It has been known for a long time that some antibodies can cross-neutralize infection by multiple HIV strains. More recently, novel technologies to investigate the B-cell repertoire have allowed the isolation of several broadly neutralizing human monoclonal antibodies resulting from natural infection. These antibodies are characterized by a high degree of somatic hypermutation compared to the germline, suggesting that their development requires long-term antigen exposure. The characterization of cross-neutralizing antibodies has led to the identification of conserved epitopes on the HIV Env protein that may be used to design new vaccines capable of conferring broader protection (reviewed by Corti & Lanzavecchia, 2013; Kwong *et al*, 2013). Furthermore, recent studies have demonstrated the therapeutic potential of passive administration of combinations of neutralizing monoclonal antibodies in the control of chronic SHIV infection in a rhesus monkey model (Barouch *et al*, 2013; Shingai *et al*, 2013). These findings suggest that a vaccine capable of eliciting cross-neutralizing antibodies targeting different epitopes on the Env trimer may control viremia in chronically infected HIV patients. Nonetheless, the design of an immunogen capable of eliciting HIV cross-neutralizing antibodies still presents considerable challenges, in particular when considering the hypermutation of the human antibodies discovered so far.

### Malaria

Approximately 250 million clinical cases of malaria are reported every year and with almost one million deaths occurring in sub-Saharan Africa mostly among children (WHO: http://www.who.int/malaria/world_malaria_report_2011/en).

A robust pipeline of malaria vaccine candidates in various preclinical and clinical phases of development is illustrated in the WHO table of vaccines (http://www.who.int/vaccine_research/links/Rainbow/en/index.html). The majority of these vaccines are based on recombinant proteins, and more than half consist of a single antigen. *Plasmodium falciparum*, the causative agent of malaria, has a complex life cycle, and while numerous antigens could feasibly be targets of protective responses at distinct phases during the cycle, these antigens are often polymorphic.

The candidate RTS,S/AS01 is the most advanced and has started the largest phase III malaria vaccine trial to date. RTS,S combines a portion of the circumsporozoite protein, the surface protein that helps the parasite invade human liver cells, with the hepatitis B surface antigen and also includes the adjuvant AS01 to further improve the immune response. In a phase II study, RTS,S/AS01 showed 53% efficacy against first malaria episode in 5- to 17-month-old children (Bejon *et al*, 2008). However, the efficacy of the vaccine was of limited duration and was not detectable 3 years after vaccination (Olotu *et al*, 2011; Bejon *et al*, 2013). The first results of the phase III trial confirmed a 55% protection in the 5- to 17-month age group. However, a lower vaccine efficacy (34.8%) was observed when 6- to 12-week-old children were included in the analysis, suggesting an age-dependent differential immune response to the vaccine (Agnandji *et al*, 2011). Final results are expected in 2014, but results so far suggest that in the target age group for whom RTS,S is intended, the efficacy against severe malaria is low. Modeled estimates of the benefits of implementing RTS,S/AS01 through routine infant immunizations predict that this vaccine could nonetheless have an impact in saving a significant number of lives (Brooks *et al*, 2012). Although the vaccine has shown mediocre efficacy and its effect declines over time, it is still expected to become the first malaria vaccine to receive regulatory approval (Bouchie, 2013). The focus for vaccine developers now moves to the next generation of malaria vaccines, but it is not yet clear what characteristics these new vaccines should have or how they can be evaluated. The understanding of the immune correlates with the aid of developments in the field of basic human immunology and systems biology may provide essential information to improve the performance of RTS,S and to fully optimize other vaccine candidates.

### Tuberculosis (TB)

The bacille Calmette-Guérin (BCG) vaccine, one of the first vaccines to be developed (Calmette *et al*, 1927), has been administered to more than 4 billion subjects thus far. Yet, *Mycobacterium tuberculosis* is responsible for more human deaths than any other single pathogen today (Ottenhoff & Kaufmann, 2012), with nearly 9 million new cases and 1.7 million deaths annually (Lawn & Zumla, 2012). The BCG vaccine is effective in infants against severe tuberculosis (TB) disease, but immunity wanes over time and BCG is not effective as a booster.

Control of TB requires a T-cell immune response and it has proven challenging to develop novel effective vaccines. Approximately 12 TB vaccine candidates are currently being evaluated in clinical trials, all of them designed to prevent active TB disease (Kaufmann, 2012). These vaccines are either (i) live recombinant mycobacteria vaccines, genetically engineered for increased efficacy and/or safety that aim to substitute BCG, or (ii) adjuvanted proteins or viral vector expressing antigens that aim to boost the immune response induced by a BCG prime.

The most advanced of the TB vaccine candidates are viral vector vaccines that are being tested in phase IIb efficacy trials. However, the current generation of vaccine candidates does not fulfill expectations. Recent results of the first efficacy trial in infants, using a modified vaccinia Ankara virus expressing antigen 85A, MVA85A, showed that the vaccine candidate was safe, but did not provide significant protection against TB when given as a booster to infants who had received BCG at birth (Tameris *et al*, 2013). Another approach based on an adjuvanted recombinant protein antigen, M72/AS01, was well tolerated and immunogenic in a phase I trial (Leroux-Roels *et al*, 2013) and is currently being assessed in phase II trials.

Next-generation vaccines should be designed to induce sterilizing immunity (Kaufmann, 2010) With the current tools available to vaccine developers such as potent adjuvants or recombinant vectors (either recombinant mycobacteria or heterologous viral carriers) and use of heterologous prime-boost combinations, a more effective TB vaccine may indeed be possible.

### Other infectious diseases (*S. aureus*/dengue virus)

While showing somewhat promising results in animals and early clinical trials, recent clinical trials of vaccines against *S. aureus* and dengue virus have given disappointing results due to the lack of efficacy and safety concerns. The first *S. aureus* vaccine tested in humans, containing types 5 and 8 capsular polysaccharides conjugated to non-toxic recombinant *Pseudomonas aeruginosa* exotoxin A (StaphVAX), appeared to confer limited short-term protection against bacteremia in hemodialysis patients (Shinefield *et al*, 2002), but in a larger phase III clinical trial failed to demonstrate significant efficacy (Jansen *et al*, 2013).

More recently, the V710 vaccine, consisting of a single highly conserved *S. aureus* antigen (IsdB), was shown to be immunogenic in healthy adults and in patients undergoing chronic hemodialysis (Harro *et al*, 2010, 2012; Moustafa *et al*, 2012). An increase in specific anti-IsdB IgG titers was observed postvaccination and maintained for 1 year in hemodialysis patients. However, the subsequent large phase IIb/III study to evaluate the efficacy and safety of preoperative vaccination in patients undergoing cardiothoracic surgery was interrupted as vaccination did not reduce the rate of serious postoperative *S. aureus* infections (Fowler *et al*, 2013).

To date, *S. aureus* vaccine candidates have been designed to elicit antibody production against one antigenic component of the bacterium; however, protective immunity against *S. aureus* is not yet understood. The failures of passive immunization strategies in clinical trials (Schaffer & Lee, 2008; Ohlsen & Lorenz, 2010; Otto, 2010) suggest that humoral immunity may be important but insufficient to prevent *S. aureus* infections. Furthermore, patients with quantitative and qualitative T-cell or neutrophil disorders have increased susceptibility to recurring *S. aureus* infections, suggesting that cell-mediated immunity and in particular Th17 responses may play an important role (Proctor, 2012; Spellberg & Daum, 2012). Current work focusing on understanding correlates of protection for *S. aureus* in humans will serve the development of next-generation vaccines. Such vaccines should preferably combine antigens that stimulate humoral and cellular responses against *S. aureus*.

Dengue fever is a complex flaviviral disease that is caused by four antigenically distinct dengue viruses (DENV-1, 2, 3, and 4) and infects 50–100 million people per year with no therapy or vaccine currently available. The dengue virus presents a particular conundrum to vaccine development due to the safety concerns associated with the potential increase in the rate or severity of dengue disease by an incomplete immune response associated with poorly protective or low neutralizing antibody levels against the four serotypes (Halstead, 1988). Thus, the goal of dengue virus vaccine development is to produce a balanced protective antibody response against all four dengue virus (DENV) serotypes and to avoid an incomplete immune response that theoretically could facilitate pathogenesis. Currently, several kinds of dengue virus vaccines are in development but only one, consisting of four recombinant live-attenuated chimeric yellow fever-based dengue virus (CYD), has reached the late stages of clinical efficacy trials (Heinz & Stiasny, 2012).

In a recent phase IIb trial, disease incidence of DENV3 and DENV4 serotypes was reduced by 80–90% upon vaccination with the tetravalent CYD vaccine. Disease caused by DENV1 was also reduced albeit to a lesser extent, while there was no efficacy against DENV2, which was the most prevalent serotype causing infections in the study (Sabchareon *et al*, 2012). CYD-2 monovalent DENV2 vaccine showed excellent immunogenicity in a phase I trial (Guirakhoo *et al*, 2006); however, neutralizing titers were lower in the tetravalent formulation in monkeys and previous human studies (Guy *et al*, 2009; Morrison *et al*, 2010; Poo *et al*, 2010). In light of results from the phase IIb study (Sabchareon *et al*, 2012), showing high levels of neutralizing anti-DENV2 antibodies, the lack of protection against DENV2 was surprising. The authors suggested that a novel DENV2 genotype circulating within Thailand had diminished cross-reaction with the elicited anti-DENV2 antibodies. However, the results have also been interpreted as evidence of possible viral interference in this trial (Halstead, 2012; Swaminathan *et al*, 2013). The vaccine has now been administered to more than 6,000 children and adults from dengue endemic and non-endemic areas and no severe disease in the trial participants has been reported. However, safety and efficacy are inextricably linked for dengue virus vaccines. The theoretical risk of vaccine-related adverse events, such as immune enhancement of infection, necessitates that long-lasting protective immune responses against all four dengue serotypes should be simultaneously induced.

## Vaccine target populations

Our society progressively sees a lower proportion of children and young people and a higher proportion of elderly people. The increase in life expectancy during the 20th century is mainly associated with reductions in infectious disease mortality in children, largely due to vaccination, and decreases in old-age mortality due to new therapies and several other factors, including reduced lifetime exposure to inflammation (Finch & Crimmins, 2004; Rappuoli *et al*, 2011). While the majority of the vaccines currently available have been developed as pediatric vaccines, today's society clearly has quite different medical needs. Vaccination represents a potential key primary prevention for diverse age and target groups including adults and the elderly, adolescents, pregnant women, people suffering from chronic and immune-compromising diseases (Fig 2).

Senescence of the immune system makes the elderly more vulnerable to infections, and waning vaccine responses may require regular booster vaccinations. As life expectancy increases, major causes of infection and death shift from childhood diseases to infectious or non-infectious chronic illnesses in adulthood. Infections from nosocomially acquired antibiotic-resistant bacteria are most frequent in the elderly age group and would be desirable to be prevented by vaccination. Responsiveness to vaccines may be reduced in the elderly, due to their aging immune system, and formulation with adjuvants or other strategies for amplification of immune responses may be required. In addition, anti-cancer strategies could target adults and the elderly through vaccination against the causative agents of diverse infection-associated tumors such as HBV, HPV, and *Helicobacter pylori* (Rupnow *et al*, 2009; Pineau & Tiollais, 2010; Romanowski, 2011) or through novel therapeutic vaccines against self-antigens overexpressed in colon, breast, or prostate cancers. Indeed, the first decade of the 21st century saw novel prophylactic and therapeutic cancer vaccines being licensed (Siddiqui & Perry, 2006; Keam & Harper, 2008; Plosker, 2011).

Maternal vaccination can simultaneously protect the mother, her developing fetus, and the newborn in the first months of life through placental antibody transfer. Today, young women are less exposed to infectious agents or may have suboptimal responses (HIV-positive mothers) (Jones *et al*, 2011). This means that newborns are often inadequately protected via maternal antibodies, against a variety of pathogens, including CMV, influenza virus, GBS, HBV, meningococcus types A, B, C, Y, and W135 (mainly B and C in Europe), *Bordetella pertussis*, RSV, rotavirus, and tetanus. The success and safety of maternal vaccination against tetanus, influenza, and pertussis, recommended for use during pregnancy, has been recently shown (Roper *et al*, 2007; Zaman *et al*, 2008; Demicheli *et al*, 2013). And while live-attenuated vaccines such as rubella, influenzae, and yellow fever are contraindicated during pregnancy due to potential complications of the attenuated agent reaching the fetus, studies completed on the inadvertent immunization during pregnancy have not detected any adverse events (Nasidi *et al*, 1993; Castillo-Solorzano *et al*, 2011; Moro *et al*, 2011). A number of additional maternal vaccines are also in the pipeline, which could be used to combat neonatal infection such as GBS and RSV (Healy, 2012).

Travelers face exposure to many infections encountered abroad for which vaccination offers protection. There is a significant need for effective vaccines against dengue fever, cholera, ETEC, malaria, shigella, and paratyphoid fever, for which no vaccines or suboptimal vaccines are available. Furthermore, in developing countries where more than 1.5 million children die from vaccine-preventable diseases every year, effective vaccines are often not available. In addition to the need for vaccines against malaria, tuberculosis, and HIV for low-income countries, there are the so-called neglected tropical diseases, including hookworm infection, dengue fever, schistosomiasis, leishmaniasis, and non-typhoid salmonellosis, for which a new generation of ‘anti-poverty vaccines’ will be required. A number of initiatives have been launched to address both the availability of present vaccines and the development of vaccines for neglected diseases (reviewed in Rappuoli *et al*, 2011).

People with chronic diseases, such as autoimmune diseases, immunosuppressive disorders as well as people affected with HIV and individuals with chronic respiratory or cardiac disease have special vaccination needs specific to their condition. In immune-compromised subjects, live-attenuated vaccines may not be tolerated well, and inactivated or subunit vaccines, possibly with potent adjuvants, may be required to elicit protective responses.

## Enabling technologies for next-generation vaccines

**Figure 3:**
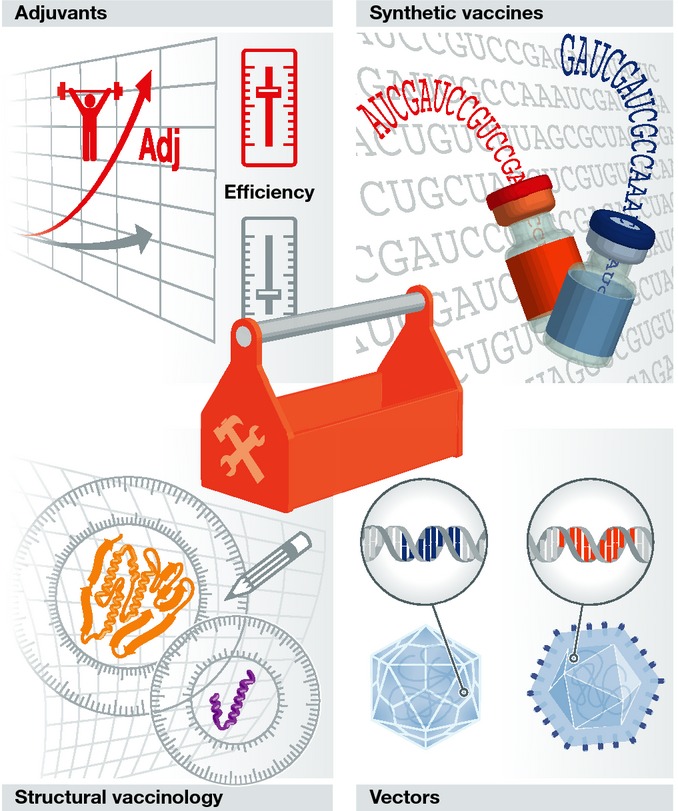
The 21st century vaccinologists toolbox

While we are struggling to develop effective vaccines against several infectious agents, progress in immunology, microbiology, genetics, and structural biology has provided a new set of tools to approach next-generation vaccine development (Fig 3). New technologies have greatly facilitated the identification of novel protective antigens, through either high-throughput discovery strategies or rational design. Next-generation vaccines are likely to show improvements in key areas such as the development of novel classes of vaccine adjuvants that can promote better protective humoral and cellular immune responses, the optimal presentation of antigens to the immune system in order to shape immune responses, and furthermore, the manufacture of vaccines using highly characterized, synthetic methods of production. Vaccines have become much safer, and it is now possible to develop vaccines against infectious agents or diseases that could not be effectively targeted using early vaccination methods.

### Vaccine adjuvants

The few adjuvants licensed for human vaccines, based on aluminum salts and oil in water emulsions, have been developed empirically, and their mechanism of action is only partially understood (De Gregorio *et al*, 2009). However, in recent years, the understanding of the molecular mechanisms of innate immune responses has dramatically increased, leading to the discovery of new classes of receptors such as Toll-like receptors (TLRs), Nod-like receptors (NLRs), and Rig-like receptors (RLRs) (Hoebe *et al*, 2004; Akira *et al*, 2006). These molecules have evolved to sense microbial infection and trigger an immune response adapted to the invading pathogens. Importantly, the innate immune reactions triggered by these receptors are also required to enhance and modulate the antigen-specific immunity. All these newly discovered innate immune receptors are ideal targets for a new generation of rationally designed vaccine adjuvants that may have a great impact on vaccine development. A large number of novel adjuvants targeting specific innate immune receptors such as TLR4 and TLR9 have been tested in human clinical trials (reviewed in De Gregorio *et al*, 2013). A few years ago, one TLR4 agonist called monophosphoryl lipid A (MPL), co-adsorbed to alum with HPV antigens, was licensed for human use (Giannini *et al*, 2006). Some of the reported effects of adjuvants in humans are: improved vaccine efficacy; increase in antibody titers and CD4 T-cell frequencies; improved duration of protective responses; increased cross-protection against different strains or variants of the same bacterial or viral pathogen; antigen dose sparing; and reduced number of doses required to reach protective antibody titers. In addition, adjuvants can modulate the quality of antibody (isotypes) and the T-cell (Th1; Th2; Th17) responses, triggering an immunity tailored to the pathogen. The new adjuvants have the potential to improve the efficacy of existing vaccines and of novel preventive and therapeutic vaccines addressing unmet medical needs.

Preclinical and human studies have demonstrated that different adjuvants can synergize if combined in the same vaccine formulation, making adjuvants even more attractive for vaccine development. For example, the AS01 adjuvant used in the RTS-S malaria vaccine described above is made by a mix of liposomes, a saponin called QS21 and MPL. However, special attention must be dedicated to the safety profile of novel adjuvants. In fact, all agonists of innate receptors are potentially toxic and must be administered in a way that optimizes adjuvanticity but reduces local and systemic reactogenicity. These two characteristics of adjuvants are likely intrinsically linked and must be carefully balanced.

### Vectors

Viral vectors, such as those based on adenoviruses or pox viruses (de Cassan & Draper, 2013), mimic a live infection by expressing antigens *in situ* after immunization, thereby facilitating the induction of strong T-cell responses, including cytotoxic T lymphocytes. These types of responses are desirable for intracellular and highly variable pathogens, as in addition to targeting the pathogen-infected cells (versus the pathogen itself), they can target epitopes that are conserved between different strains (Liu, 2010).

A broad spectrum of replicating and non-replicating vectors is available. A variety of attenuated viruses have been employed as vectors including vaccinia and other pox viruses, adenovirus, and single-stranded RNA virus replicon vectors such as alphaviruses, coronaviruses, picornaviruses flaviviruses, influenza viruses, rhabdoviruses, and paramyxoviruses. Viral vectors have undergone extensive preclinical assessment for a wide spectrum of diseases and have been tested in numerous clinical trials, and these studies have revealed hierarchies of potency for individual vectors and each viral vector has its own advantages, limitations and range of applications (reviewed in Rollier *et al*, 2011). Choice of an appropriate vector for use in the development of a vaccine depends on the biology of the infectious agent targeted, whether the vaccine is intended to prevent infection or to boost immunity in already-infected individuals, prior exposure of the target population to the vector, the number and size of gene inserts needed, and suitability for large-scale manufacturing and compliance with regulatory requirements.

Licensing of several veterinary viral vector vaccines (Poulet *et al*, 2007; Weyer *et al*, 2009) highlights the potential of this technology; however, there is still no recombinant virus vector vaccine licensed in humans. The reasons for this include limitations in potency due to pre-existing anti-vector immunity and concerns about safety as already discussed for the recombinant Ad5 vector in the HIV trials. In addition to applications in infectious disease, viral vectors have been employed for cancer vaccines and several clinical trials show encouraging results. One of the most advanced approaches is based on a prime-boost immunization using two different viral vectors (vaccinia virus and fowlpox, respectively) expressing a prostate cancer antigen (PSA) and three different co-stimulatory molecules (B7-1, LFA-3, and ICAM1). This vaccination strategy has demonstrated an increase in median survival and a 44% reduction in death rate in metastatic castration-resistant prostate cancer patients in phase II trials (Kantoff *et al*, 2010).

There is also interest in the use of live-attenuated bacteria, usually *Salmonella* or *Listeria* spp., as vectors for the presentation of heterologous antigens. Such vaccines allow immunization through the mucosal route and specific targeting of professional antigen-presenting cells located at the inductive sites of the immune system. Both humoral and cellular immune responses can potentially be primed by this approach. A further novel approach exploits intracellular bacteria as delivery vectors for DNA vaccines (Toussaint *et al*, 2013).

### Synthetic vaccines

A key advancement in synthetic vaccinology has been the use of nucleic acid-based vaccines, which combine the advantages of *in situ* expression of antigens with the safety of subunit vaccines. Vaccines based on DNA or RNA are not inhibited by pre-existing anti-vector immunity like in the case of viral vectors. The manufacturing of nucleic acid-based vaccines also offers the potential to be relatively simple and inexpensive. For about 20 years, most of the attention was focused on DNA vaccines, which have been shown to be potent in a wide variety of animal species, and several products are now licensed for commercial veterinary use (Draghia-Akli *et al*, 1997; Davis *et al*, 2001; Garver *et al*, 2005; Grosenbaugh *et al*, 2011). In humans however, while showing much promise in preclinical models, DNA vaccines have shown reduced and disappointing potency in the clinic (reviewed in Ferraro *et al*, 2011). This was likely due to poor delivery of the vaccine DNA into human cells and insufficient stimulation of the human immune system. The latest generation of DNA vaccines may rely on improved delivery either through the use of electroporation (Sardesai & Weiner, 2011) or through co-administration of genes encoding immunostimulatory cytokines (Lori *et al*, 2006; Flingai *et al*, 2013) to overcome these limitations. Recently, electroporation of a DNA vaccine encoding HPV antigens induced good antibody and CD8 T-cell responses exhibiting cytolytic functionality in humans (Bagarazzi *et al*, 2012). Furthermore, in mixed regimen immunizations, DNA vaccines can be effective in priming B- and T-cell responses. Early studies have revealed that the potency of the T-cell responses was enhanced when an initial immunization with plasmid DNA was followed by a viral vector, both encoding the same antigen in a so-called heterologous prime-boost regimen in that it was more potent than either the DNA or viral vector alone, independently of the order of administration (Schneider *et al*, 1998). More recently, heterologous prime-boost regimens mainly use a viral vector or a DNA vaccine for priming, followed by a boost with a protein-based vaccine. For example, in the prime-boost strategy of the RV144 study, subjects were primed with a canarypox vector encoding gag, env, and protease and boosted with a gp120 subunit vaccine (Rerks-Ngarm *et al*, 2009). This immunization schedule results in the induction of a strong cellular immune response and is associated with a higher and more specific antibody response against the vaccine target compared to homologous immunization and can overcome the issue of anti-vector immunity.

RNA vaccines, based on mRNA or RNA replicons, may offer certain advantages over plasmid DNA and viral vectors. RNA vaccines are active in the cytoplasm, do not require delivery to the nucleus, and therefore avoid the potential issues of DNA integration. However, the increased susceptibility to degradation of RNA compared to DNA has required additional stabilizing technologies (Geall *et al*, 2013). To date, several exploratory trials in cancer patients with mRNA vaccines have resulted in the induction of anti-tumor immunity, demonstrating proof of concept in humans (Weide *et al*, 2008, 2009; Rittig *et al*, 2011). RNA vaccines can also be engineered RNA replicons derived from certain RNA viruses lacking viral structural proteins which are capable of self-replication on delivery to the cytoplasm (Geall *et al*, 2012, 2013). The RNA amplification process leads to double-stranded RNA intermediates, which are known to be potent stimulators of innate immunity and therefore may have inherent adjuvanticity with respect to mRNA vaccines. As with DNA vaccines, formulation and enabling delivery technologies will be an important area of research for RNA vaccines.

A recent publication reports the collaborative efforts to develop a rapid process for synthetic vaccine virus generation in one of the first real-world products from synthetic biology (Dormitzer *et al*, 2013). While influenza vaccine preparations have been administered to humans since the mid-1930s, the challenges within this field have continued to drive advances in technologies and the development of new approaches. In this recent study, three major technical barriers for a rapid and reliable response to pandemic flu were addressed: the speed of synthesizing DNA cassettes to drive production of influenza RNA genome segments, the accuracy of rapid gene synthesis, and the yield of HA from vaccine viruses. The implementation of synthetic seed generation in influenza vaccine manufacturing would enable high-yielding vaccine virus availability to manufacturers for testing, scale-up, and process optimization: within days, not months, after a new virus is first detected.

### Structural vaccinology

Detailed three-dimensional (3D) structure, domain organization, and dynamics of surface proteins of pathogens offer molecular targets that can guide the design of effective vaccines and better immunogens by stabilizing native conformations or combining, exposing, and/or improving the immunogenicity of epitopes (reviewed in Back & Langedijk, 2012; Burton *et al*, 2012; Kulp & Schief, 2013). Important goals of structural vaccinology are to stabilize a conformation of an antigen capable of eliciting protective responses or to selectively present the conserved determinants of complex and variable antigens in order to focus immune response to conserved epitopes.

The F protein of RSV is a major target of structure-based vaccine design. The F glycoprotein adopts two conformations on the virus: prefusion (before infection) and postfusion (after infection) which are both recognized by neutralizing antibodies (reviewed in McLellan *et al*, 2013c). The determination of the 3D structure of the postfusion state of the RSV F glycoprotein has allowed the engineering of a more stable F immunogen able to elicit neutralizing antibodies (Swanson *et al*, 2011). More recently, elucidation of the crystal structure of the prefusion state of the RSV F in complex with a neutralizing antibody (McLellan *et al*, 2013b) paved the way for the structure-based design of the first stable prefusion F antigen with superior immunogenicity when compared to the postfusion antigen (McLellan *et al*, 2013a).

One potential application of structural vaccinology is the design of an improved antigen to prevent HIV infection. The Env protein (heterodimer made up of gp41 and gp120, natively present in trimers) is the sole target of HIV neutralizing antibodies. However, due to the instability of the trimer in solution and the immunodominance of the variable regions, it has been the candidate for many structural studies in rational immunogen design (reviewed in Burton *et al*, 2012). Approximately 20% of HIV-infected individuals develop broadly neutralizing antibody (bNAb) responses over time, and over the last 2 years, many of the relevant epitopes have been defined and mapped through the use of novel technologies (reviewed in Corti & Lanzavecchia, 2013).

These findings serve to identify highly conserved and invariant structures as targets for bNAbs that can serve for rational immunogen design through various approaches. Integrating structure and sequence information for families of bNAbs has recently enabled the creation of germline-targeting immunogens that bind and activate germline B cells in order to initiate the elicitation of such antibodies (Jardine *et al*, 2013). Although no bNAb responses have successfully been elicited by HIV vaccine candidates to date, the finding in the RV144 trial that antibody responses could contribute to protection (Rerks-Ngarm *et al*, 2009; Haynes *et al*, 2012) is encouraging. Furthermore, the recent elucidation of the crystal and cryo-EM structures of the Env trimer (Julien *et al*, 2013; Lyumkis *et al*, 2013) will open a new paragraph in structure-based design for next-generation improved HIV-1 immunogens.

### Human immunology

A critical question for the success of vaccines in the future is which technology, alone or in combination, must be used to elicit a protective response. The answer will not be the same for different pathogens and will be based on the integration of different immune effector mechanisms of the appropriate quality. To this end, it will be important to assess the impact of novel vaccine technologies in human trials and correlate multiple immune readouts with protection. The progress in genomics allows the generation of huge amounts of high-throughput data from human blood samples including RNA and protein expression profiles, B-cell repertoire analysis, single cell analysis, and analysis of genomic polymorphism. Systems biology is required to interrogate the genomic data and identify molecular signatures which correlate with the immunological analyses obtained from the same subjects through classical immunological assays for antibodies and T-cell characterization (Pulendran *et al*, 2010). In studies of vaccine responses with the yellow fever and influenza vaccine, these approaches have already been successfully applied leading to the identification of ‘protective signatures’ to predict immunogenicity of the vaccine in human subjects (Gaucher *et al*, 2008; Querec *et al*, 2009). The final goal of using systems biology to interrogate human vaccine responses is to identify biomarkers of safety and efficacy. These vaccine biomarkers have the potential to accelerate the time of vaccine development, allowing for the selection of the most promising vaccine candidates in early exploratory clinical trials, before proceeding to long, expensive efficacy trials that involve a very large number of subjects.

## Conclusions

The beginning of the 21st century has already seen new vaccines licensed and become available due to the development of novel approaches. Novel technologies, such as the virus-like particles, have allowed the development of vaccines against HPV (Siddiqui & Perry, 2006; Keam & Harper, 2008). Reverse vaccinology, through mining of genome sequences for high-throughput antigen discovery, has successfully allowed the development of a novel multicomponent recombinant vaccine against meningococcus type B (Giuliani *et al*, 2006). The first therapeutic vaccine based on blood cell infusion has been licensed for prostate cancer (Plosker, 2011). Several tools have been developed and in some cases already tested in human trials, which will greatly support the discovery and rational design of novel vaccines against difficult targets such as HIV, malaria, TB, dengue, and *S. aureus*, where conventional technologies have failed. The hope is that, thanks to these technologies, more infectious diseases will be preventable by vaccinating children, adolescents, adults and elderly, pregnant women, and immunocompromised subjects. Novel vectors and adjuvants may also allow the development of therapeutic vaccines to treat different forms of cancer, chronic infections, and other inflammatory disorders. The development of innovative immunization regimes and novel delivery technologies provides unprecedented means to not just augment but to shape the immune responses. What the experiences of recent clinical trials have taught us is that while the quantity or magnitude of immune responses is important, the quality or flavor of these responses is equally important, and predicting immunogenicity does not necessarily translate into predicting protection. For many of the elusive targets in vaccine development, one of the most challenging gaps to fill is that of identifying biomarkers or correlates of protection. An immediate goal we should set is to exploit the trials undertaken to date, in the attempt to identify signatures of vaccine efficacy that can guide early selection of the most promising vaccine candidates for the future. Understanding what responses are desirable and necessary for protection and how they can be induced by a vaccine will unlock the door to rationally designing effective next-generation vaccines.

**Pending issues**

Identify biomarkers that correlate with vaccine protection or safety.

Use antibody repertoire analysis to identify novel protective epitopes.

Use structural information on protective epitopes to design better immunogens.

Improve knowledge of host-pathogen interactions and mechanisms of protection.

Capture synergy of using different adjuvants or multiple vaccine technologies.

Develop dedicated vaccines for elderly, pregnant women, and infants.

Evaluate the impact of vaccines in combination with antibody-based immunotherapy.

## For more information

GIVS 2006–2015 at http://www.who.int/immunization/givs/en/

UNIAIDS Global Report at http://www.unaids.org/en

WHO report 2010: http://www.who.int

HVTN505 result update at http://www.hvtn.org/505-announcement-25April2013.html

WHO table of vaccines: http://www.who.int/vaccine_research/links/Rainbow/en/index.html

WHO: http://www.who.int/malaria/world_malaria_report_2011/en
